# Stereotactic Body Radiotherapy for Early-Stage Non–Small Cell Lung Cancer: When and Why Is It Appropriate Therapy?

**Published:** 2015-07-01

**Authors:** Kim Mullins

**Affiliations:** University of Cincinnati College of Nursing, Cincinnati, Ohio, and Oncology Hematology Care, Cincinnatti, Ohio

Lung cancer, the second most frequently diagnosed malignancy for men and women in the United States, will account for an estimated 221,200 newly diagnosed cases and 158,040 deaths in 2015 (Siegel, Miller, & Jemal, 2015). The preferred treatment for early-stage non–small cell lung cancer (NSCLC), often defined as stage I or stage II, is surgical excision (Ishikura, 2012; Kelsey & Salama, 2013; Potters et al., 2010). The 5-year overall survival (OS) rate without treatment is 20%. The OS rate for total lobectomy is 30% to 50% for patients with stage II NSCLC and 60% to 70% for patients with stage I NSCLC (Guckenberger et al., 2014; Landreneau et al., 1997; Port, Kent, & Altorki, 2002; Scott, Howington, Feigenberg, Movsas, & Pisters, 2007). It is estimated, however, that 20% to 25% of patients with lung cancer do not have surgery, either by choice or because of their comorbidities (Ishikura, 2012). The treatment options then include surveillance, chemotherapy, radiation therapy, or an ablative procedure.

Surgical excision is considered the standard of care for patients with early-stage NSCLC (Davis, Medbery, Sharma, Danish, & Mahadevan, 2013; Ishikura, 2012; Kelsey & Salama, 2013; Robinson et al., 2013; Senan, Paul, & Lagerwaard, 2013). Total lobectomy, if possible, is preferred over subtotal lobectomy (also called wedge resection) due to the likelihood of disease recurrence (Fernando & Timmerman, 2012; Kelsey & Salama, 2013). Studies have shown that sublobar resection has a local recurrence that is three times higher than that of lobectomy (Senan et al., 2013).

However, it is estimated that 20% to 40% of patients diagnosed with stage I or stage II NSCLC who do not have surgery, either by necessity or choice (Allibhai et al., 2013; Senan et al., 2013). The number of patients diagnosed with early-stage NSCLC is expected to rise, as low-dose lung computed tomography (CT) screening is now advocated and more accessible (Allibhai et al., 2013). Although this will likely result in increased numbers of patients who meet the criteria for lobectomy, there will also be an increased number of inoperable patients for whom stereotactic body radiotherapy (SBRT) will be a recommended alternative treatment.

## CONVENTIONAL RADIATION VS. STEREOTACTIC BODY RADIOTHERAPY

Stereotactic body radiotherapy is defined as a form of external radiation therapy that accurately delivers a high dose of radiation precisely to one or a few extracranial body sites that are confined to a smaller radiation field (Chan et al., 2012; Howington, Blum, Chang, Balekian, & Murthy, 2013; Potters et al., 2010). Sahgal and colleagues (2012) further described SBRT as intended to provide long-term control. To accomplish this, there are certain technical requirements that must be met.

Onishi and Araki (2013) stated the four conditions for SBRT: (1) stability and reproducibility of the treatment plan; (2) measures in place to correct or prevent respiratory movement error; (3) dose concentration onto the tumor by multidirectional three-dimensional coverage; and (4) a short treatment period. Other considerations include the size (< 4 cm) and location of the lung tumor (Allibhai et al., 2013). The most common extracranial site of SBRT is the lung (Davis et al., 2013; Howington et al., 2013; Sahgal et al., 2012).

There are several benefits to SBRT. It does not require any anesthesia, there are no risks associated with the operating room, there is no surgical incision, the treatments can be completed in a series of 1 to 5 fractions over 1 to 2 weeks, there is no recovery time, lung function is minimally impacted, and there is less of a chance of missed margins than in surgery (Howington et al., 2013; Timmerman et al., 2006). The risks associated with SBRT are variable, depending on where the tumor is located and what normal tissue resides around that space. Potential risks include pulmonary toxicity, chest wall and/or skin toxicity, esophageal fistula, rib fracture, chest wall pain syndrome, or brachial plexopathy (Kelsey & Salama, 2013). High-grade pulmonary toxicity is more likely in larger or more central tumors. In addition, chest wall pain or rib fractures were found to be more likely (30%) as the volume of chest wall exposed to 30 Gy or more increased (Kelsey & Salama, 2013).

When comparing the side effects of central vs. peripheral tumors, a prospective single-facility analysis found that there was no statistical difference in side effects (Mangona et al., 2015). The most common side effects noted at 2 years were grade > 2 pain (14% central, 19% peripheral), musculoskeletal complaint (5% central, 10% peripheral), pneumonitis (6% central, 10% peripheral) and skin concerns (10% central, 3% peripheral; Mangona et al., 2015).

Conventional radiation, utilized for more than 30 years, consists of daily treatments, Monday through Friday, for 6 to 8 weeks for NSCLC (Kelsey & Salama, 2013). Research on inoperable patients with NSCLC has found that this type of radiation therapy provides a 5-year OS of 6% to 27% (Ishikura, 2012). With conventional radiation to the lungs, small doses (5 days a week) of radiation are required to protect the normal tissue exposed to radiation during treatment. Treating a large area with high doses of radiation would be too toxic for patients. Within the past 2 decades, SBRT has become more popular due to the technical ability to deliver very high doses of radiation to smaller, confined areas over short periods (Guckenberger et al., 2013; Kelsey & Salama, 2013; Potters et al., 2010).

Stereotactic body radiotherapy is preferred over conventional radiation therapy for the treatment of early-stage, inoperable lung cancer due to less local tumor relapse (Guckenberger et al., 2013; Kelsey & Salama, 2013; Iyengard, Westover, & Timmerman, 2013). Kelsey and Salama (2013) reported that conventional radiation treatment of lung cancer has a recurrence rate that is 25% to 50% higher than SBRT. In a Japanese phase II study, Ishikura (2012) found that the rate of OS for patients with NSCLC receiving SBRT was 56% at 3 years, and the rate of local tumor control at 3 years was 85% to 95%.

Many other studies have shown that SBRT is superior to conventional radiation for treatment of inoperable early-stage NSCLC (Howington et al., 2013; Kelsey & Salama, 2013; Senan et al., 2013; Timmerman et al., 2006). There are studies emerging in which SBRT shows nearly equivalent rates of local control as surgical lobectomy but without the toxicity or mortality risk as surgery (Grills et al., 2010; Senan et al., 2013; Timmerman et al., 2006).

## FACTORS AFFECTING SBRT LOCAL CONTROL

Many factors have been evaluated for their possible effect on the rates of local control with SBRT in lung cancer. Miyakawa and colleagues (2013) evaluated whether histology played a role in tumor control by SBRT. They found that although squamous cell carcinomas initially showed a more rapid radiologic response, by 6 months posttreatment, there was no significant difference between these carcinomas and adenocarcinomas treated in the same manner.

Several authors have found that SBRT is less effective on large tumors (> 4 cm; Allibhai et al., 2013; Chan et al., 2012; Howington et al., 2013). Radiation dose has emerged as a factor showing the greatest statistically significant impact on tumor control. There is a proven SBRT dose-response relationship to local control, suggesting some dosing schemas are more likely to achieve higher rates of local control (Guckenberger et al., 2014). The typical dosing schedules may vary by region. For example, Dahele et al. (2008) looked at the literature and found the most common dose reported in the United States was 54 to 60 Gy in 3 fractions, compared with 48 Gy in 4 fractions given in Japan and 60 Gy in 5 to 8 fractions given in Europe.

However, it has been found that an aspect of the radiation dose—referred to as the biologically effective dose (BED)—may be a better indicator of outcome than dose alone (Allibhai et al., 2013; Dahele et al., 2008; Guckenberger et al., 2014). The BED is a measure of the true biologic radiation dose delivered to a particular tissue, which takes into account the dose per fraction, days to complete therapy, and the total dose. This formula considers not only the dose the tissue received but also the cellular repair that can occur between treatments. The BED is a calculation that compares treatment regimens to quantify the radiation dose necessary to provide tumor kill.

Guckenberger et al. (2014) stated that the BED is the single most predictive factor affecting local control with SBRT and OS. They go on to state that a BED of greater than 106 Gy results in local tumor control of 92.5% and OS of 62% at 3 years (Guckenberger et al., 2014). As a point of reference, it is reported that the BED for SBRT given as 48 Gy in 4 fractions is 105 Gy; 60 Gy given in 5 to 8 fractions is 132 Gy; and 60 Gy given in 3 fractions is 180 Gy. In contrast, the conventional external-beam radiation dose of 70 Gy given in 35 fractions results in a BED of 84 Gy (Dahele et al., 2008). This comparison may help to explain the increased recurrence rates of conventional lung radiation compared with SBRT.

## CONCLUSION

Stereotactic body radiotherapy is a form of radiation therapy used to treat patients with NSCLC who do not have surgery, whether by choice or necessity due to comorbidities (Guckenberger et al., 2014; Kelsey & Salama, 2013). Stereotactic body radiotherapy to the lungs is well tolerated and carries less mortality risk than surgical intervention.

Another benefit of SBRT treatment of NSCLC is a high rate of local tumor control. Various studies have reported local control rates in the realm of 80% to 100% (Allibhai et al., 2013; Guckenberger et al., 2014; Ishikura, 2012; Onishi & Araki, 2013). Although surgical lobectomy remains the gold standard for patients with early-stage NSCLC, SBRT to the lungs is a recommended treatment alternative for nonsurgical candidates. Given the emerging evidence showing that the rates of local control and OS of SBRT are approaching those of lobectomy for early-stage NSCLC, we may see SBRT join surgery as a first-line treatment option in the future.
